# Study of Serum Copper and Zinc Levels and Serum Cu/Zn Ratio among Polish Women with Endometrial Cancer

**DOI:** 10.3390/nu16010144

**Published:** 2023-12-31

**Authors:** Katarzyna Kluza, Izabela Zawlik, Magdalena Janowska, Aleksandra Kmieć, Sylwia Paszek, Natalia Potocka, Marzena Skrzypa, Alina Zuchowska, Marta Kluz, Andrzej Wróbel, Piotr Baszuk, Sandra Pietrzak, Wojciech Marciniak, Pawel Miotla, Jan Lubiński, Jacek Gronwald, Tomasz Kluz

**Affiliations:** 1 Department of Gynecology, Gynecology Oncology and Obstetrics, Fryderyk Chopin University Hospital, F. Szopena 2, 35-055 Rzeszow, Poland; katarzyna.jaworska.m@gmail.com (K.K.); aleksandra.kmiec@vp.pl (A.K.); 2 Laboratory of Molecular Biology, Centre for Innovative Research in Medical and Natural Sciences, Medical College of Rzeszow University, Warzywna 1a, 35-959 Rzeszow, Polandnpotocka@ur.edu.pl (N.P.);; 3 Institute of Medical Sciences, Medical College of Rzeszow University, Kopisto 2a, 35-959 Rzeszow, Poland; alzuch4@gmail.com; 4 Department of Pathology, Fryderyk Chopin University Hospital, F. Szopena 2, 35-055 Rzeszow, Poland; marta.kluz@interia.pl; 5 Second Department of Gynecology, Medical University of Lublin, Jaczewskiego 8, 20-954 Lublin, Poland; wrobelandrzej@yahoo.com (A.W.);; 6 Department of Genetics and Pathology, International Hereditary Cancer Center, Pomeranian Medical University in Szczecin, Unii Lubelskiej 1, 71-252 Szczecin, Poland; 7 Read-Gene, Grzepnica, Alabastrowa 8, 72-003 Dobra, Poland

**Keywords:** microelements, cancer, copper, zinc, endometrial cancer

## Abstract

Background: Micronutrients are important components for the homeostasis of the human body. The studies available in the literature of the subject on their impact on the risk of population diseases, including malignant neoplasms, are ambiguous. In this paper, the relationship between Cu and Zn serum levels and the occurrence of endometrial cancer have been analyzed. Methods: 306 patients (153 test group and 153 control group) matched for age were analyzed for Cu and Zn levels. Microelements levels were determined for sera collected during the hospitalization of patients by means of an inductively coupled plasma mass spectrometry. In addition, the Cu/Zn ratio in the population included in the study was analyzed. Univariable and multivariable analyzes were used to examine the relationship between the factors under study and the incidence of endometrial cancer. Results: Lower levels of elements were observed in the study group compared with the control group (Cu: 959.39 μg/L vs. 1176.42 μg/L, *p* < 0.001; Zn: 707.05 μg/L vs. 901.67 μg/L, *p* < 0.001). A statistically significant relationship with the occurrence of endometrial cancer was observed for Cu and Zn. The patients with the lowest Cu level had a significantly higher occurrence of endometrial cancer compared with reference tertile (OR 8.54; *p* < 0.001). Similarly, compared with the reference tertile, the patients with the lowest Zn levels had a significantly greater incidence of endometrial cancer (OR 15.0; *p* < 0.001). Conclusion: The results of the study suggest an association of endometrial cancer occurrence with lower Cu and Zn serum levels.

## 1. Introduction

Copper (Cu) and zinc (Zn) are two of the most important micronutrients for the human body. They affect many processes in the human body as they act as a structural ion, catalyst, and regulator of enzymatic reactions; they participate in antioxidant processes, immune response, aging processes, and have anti-inflammatory effects [1,2,3,4,5,6]. Both are important for the structure and function of many enzymes, among others dehydrogenases, aldolases, peptidases, phosphatases, and dismutases such as the Cu-Zn SOD (superoxide dismutase) [1]. Their content in the body depends, inter alia, on lifestyle, age, or environmental influence [7]. Changes in Cu and Zn levels in the body have been implicated in various disease states. Because of a wide range of functions in the human body, the role of Cu and Zn in the process of carcinogenesis is also of interest.

Zn is considered the most important micronutrient in the human body. It is essential as a structural or functional element for at least 3000 proteins [8]. It is involved in DNA synthesis, RNA transcription, and cell division of the immune response [3,6,8,9,10,11,12,13,14,15]. The content of Zn in the body is estimated at about 2–4 g [16], of which only 0.1% is contained in the plasma (plasma concentration is 13.8–22.9 μmol/L [15]), while the remainder is intracellular. It is estimated that about 10% of the human proteome contains Zn. Its highest levels are found in the retina and choroid, liver, bones, and skin [17,18]. In plasma, Zn is bound and transported by albumin and transferrin. The distribution, absorption, and regulation of Zn activity are strictly controlled by metal transporters of Zirt-/Irt-like protein (ZIP) family and transporters of Zn transporters family (ZnT) and intracellular binding proteins—metallothienins [19,20]. The optimal daily intake of Zn in the diet is estimated at 11 mg/day for men and 8 mg/day for women [21]. Deficiency and excessive levels of Zn have an adverse effect on the human body. Groups with an increased demand for Zn include neonates, children, adolescents, pregnant women, and breastfeeding women [22]. Most common causes of Zn deficiency include eating disorders (anorexia and bulimia), eating habits (veganism), gastrointestinal diseases such as Crohn’s disease, ulcerative colitis, diarrhea, kidney disease, and diabetes mellitus [23].

Except for this, a high intake of Cu, iron, or phytic acid can also result in a malabsorption of dietary Zn [24]. The symptoms of Zn deficiency include diarrhea, weakened immunity, decreased appetite, increased secretion of pro-inflammatory cytokines, skin reactions, delayed wound healing, decreased fertility, and reduced growth [25,26].

Recently, the role of Zn in carcinogenesis has been extensively studied, but still the impact of changes in the level of this element on carcinogenesis is not fully explained. The mechanisms of Zn’s influence on carcinogenesis include changes in cell/membrane transporters, the effect on DNA transcription associated with zinc finger (ZnF) proteins, antioxidant activity, and the action of zinc-dependent matrix metalloproteinases (MMP) [27,28,29]. This ion, as an antioxidant, protects genetic material. Zn is an important cofactor of the copper–zinc superoxide dismutase (CuZnSOD), which is one of the most significant enzymes responsible for the neutralization of free oxygen radicals (ROS) [30]. ROS are well documented procarcinogenesis factors [30,31,32]. Oxidative stress damage of DNA and chromosome breaks were reported in animals with a low Zn diet [33]. Zn prevents the transformation of H_2_O_2_ to hydroxyl radicals and reduces the reactivity of sulfhydryl groups [34,35]. Zn can also weaken the ROS effect by the induction of the synthesis of methalothioneins [36]. Moreover, Zn takes part in the activation and structural stabilization of the protein p53, which is a transcription factor responsible for regulating cell life, apoptosis, and the processes of repairing the genetic material [35,37,38]. Other Zn-related transcription factors are ZnF proteins. ZnF proteins, through numerous signaling pathways, influence cell proliferation, apoptosis, migration, and the metastasis of cancer [39,40]. Therefore, Zn deficiency is connected with oxidative DNA damage and compromise DNA damage repair responses, which underlies carcinogenesis. A separate mechanism of zinc’s impact on carcinogenesis is its effect on the immune system. The effect of Zn on the immune system is important for both humoral and cellular responses. In a state of deficiency of this ion, granulocyte chemotaxis, phagocytosis, activity of monocytes, and natural killer cells, as well as cytokine production, are impaired [10,41,42]. The Th1/Th2 lymphocyte balance is also disturbed (toward Th2 activity). The above-mentioned processes are crucial for the immune fight against cancer.

Cu, despite the fact that it is present in the human body in physiological conditions in a much smaller amount than Zn, is necessary to maintain proper homeostasis. Cu is involved in many physiological processes such as cellular respiration, free radical detoxification, neuropeptide processing, cell proliferation, and angiogenesis [43,44,45]. Cu, like Zn, plays an important role in the operation of many enzymes (cytochrome c oxidase, Cu/ Zn superoxide dismutase, lysyl oxidase, and tyrosinase) [46]. Its content in the body is estimated at 75–100 mg, and the acceptable daily intake for this element is 1.6 mg/day for men and 1.3 mg/day for women [21,46]. Cu absorption occurs primarily in the small intestine [47]. Liver is the main organ regulating Cu metabolism and it acts as a storage and a distributor for other organs, and a place where Cu is excreted with bile outside the body [48]. The brain is the second organ in terms of Cu content, but small amounts can also be found in the heart, brain, kidneys, and muscles [49]. In blood circulation, Cu is bound to plasma proteins, including ceruloplasmin, albumin, and transcuprein—for transport to the organs and tissues [50]. At this point, it should be noted that the process of absorption, indirectly through methylthienins, is influenced by Zn [51]. The symptoms of Cu deficiency include hematological disorders, neurological disorders, osteoporosis, joint problems, disorders in glucose and cholesterol management, weakening of immunity, fatigue, or thyroid function disorders [52]. The toxic effect of Cu is mainly related to the generation of oxygen free radicals in redox reactions that damage DNA [53,54]. Recently, many studies have shown the impact of disturbances in Cu homeostasis on increasing the likelihood of cancer development and progression. Cu is involved in many processes related to the development and progression of cancer, such as cell proliferation, angiogenesis, and the formation of metastases [47,48,54,55]. Cu is thought to play a significant role in some of cellular signaling pathways that contribute to carcinogenesis. Cu promotes tumor growth through, among others, MAPK and MEK pathways, and it enhances protein degradation through activation of the E2 conjugating enzyme clade, including p53 protein [56,57]. In some cancer cells, Cu is also responsible for upregulation of PDL-1 dependent immune system abundance [58]. Moreover, Cu is able to stimulate angiogenesis by activating factors such as vascular endothelial growth factor (VEGF), interleukin-1 (IL-1), and tumor necrosis factor (TNF) [54,59,60]. Another point of procarcinogenic effect of Cu is the copper-dependent lysyl oxidase enzymes (LOX family), which take part in the metastasis process [57]. On the other hand, Cu can direct the cell towards apoptosis (cuproptosis) and thus act as an antitumor factor. Cuproptosis is associated with increased intracellular and mitochondrial levels of Cu ions. This leads to cell death associated with the accumulation of ROS [61].

Endometrial cancer (EC) is the most common cancer of reproductive organs in developing countries. According to GLOBOCAN, in 2020, there were 417,000 new cases and 97,000 deaths due to EC globally [62]. Along with the growing epidemic of obesity, which is the main risk factor for morbidity, the number of new cases is increasing [63]. Other EC risk factors include diabetes mellitus, hypertension, early menarche, late menopause, and insulin resistance, as well as the use of tamoxifen [64]. Major risk factors are connected to exposure to estrogens [65,66]. A vast majority of patients are symptomatic, and the first symptom is usually abnormal bleeding from the genital tract [67]. Patients with advanced disease may present symptoms such as abdominal pain, flatulence, dysuria, constipation, decreased exercise tolerance, and dyspnoea due to pleural effusion [68]. Approximately 1% to 5% of EC diagnoses occur in asymptomatic women [69]. The five-year overall survival rate for patients with EC depends on the stage at the time of the initiation of treatment and ranges from 95% for FIGO I stage to 18% for FIGO IV [70]. Currently, when assessing the prognosis of patients with EC, the molecular classification of this tumor according to The Cancer Genome Atlas (TCGA) is taken into account. There are four molecular subgroups of EC that have been distinguished: POLE mutated, microsatellite instability (MSI), copy-number low, and copy-number high. The five-year progression-free survival rate (PFS) is the best with POLE mutated EC’s and the worst with copy-number high ECs [71].

Many studies have described correlations between the levels of trace elements (Zn, Cu, and Se) and the risk of malignant tumors [72,73,74,75]. These influences have been demonstrated for prostate [76], breast [77], lung [78], ovary [79], and colorectal cancer [80]. In addition to studies that analyze the effect of levels of individual micronutrients on the occurrence of cancer, there are studies on the relationship of trace elements (ratio) in the context of the impact on the occurrence of malignant tumors [81,82].

The purpose of our study was to analyze the relationship between Cu and Zn levels, the Cu/Zn ratio, and the occurrence of EC.

## 2. Materials and Methods

A group of 306 women from the south-eastern Poland, who were patients of the Clinical Department of Gynecology, Oncological Gynecology, and Obstetrics of the University Hospital in Rzeszów, were enrolled to the study. The study group consisted of 153 patients with a histopathological diagnosis of EC. The control group consisted of 153 patients from the Clinical Department of Gynecology, Oncological Gynecology, and Obstetrics of the University Hospital in Rzeszów admitted due to fibroids and urinary incontinence for preoperative curettage of the uterine cavity. A negative oncological result of curettage of the uterine cavity and a lack of a current diagnosis of another neoplastic process were additional criteria for inclusion in the control group. The control group was matched with the study group based on age (±3 years). The study was conducted after obtaining the consent of the Bioethics Committee (Resolution No. 90/B/2016 of the Bioethics Committee of the Regional Medical Chamber of 24 November 2016). All patients signed written consent and completed a questionnaire regarding their health, lifestyle, and medical history. The study was conducted in accordance with the Helsinki of Declaration.

### 2.1. Sample Collection and Storage

Blood samples were collected from all patients during hospitalization. In the patients diagnosed with EC, blood was collected before the oncological surgery, and in the patients from the control group, before the procedure of curettage of the cervical canal and uterine cavity. All sera were collected with a Vacutainer^®^ System (BD, Franklin Lakes, NJ, USA). Blood for serum samples was collected into tubes containing a cloth activator. After collection, the tubes were incubated at room temperature for a minimum of 30 min to clot, and after this time, the tubes were centrifuged in 1300× *g* for 12 min. After the centrifugation serum was aliquoted and transferred into new cryovials and then deep-frozen (−80 °C) until analysis. On the day of analysis, the sera were thawed, vortexed, and centrifuged at 5000× *g* for 5 min.

### 2.2. Measurement Methodology

The inductively coupled plasma mass spectrometer ELAN DRC-e (PerkinElmer, Waltham, MA, USA) was applied to test the concentrations of Cu and Zn. Oxygen was used as the reaction gas. Before each cycle, the device was calibrated to obtain parameters consistent with the manufacturer’s data. The spectrometer was calibrated using an external calibration technique. Calibration standards were prepared fresh daily, from 10 µg/mL Multi-Element Calibration Standard 3 (PerkinElmer, Waltham, MA, USA) by diluting with a blank reagent to a final concentration of 1, 2, 5, 10, and 50 for Zn and Cu µg/L determination. Correlation coefficients for calibration curves were always greater than 0.999. Matrix-matched calibration was used. The analysis protocol assumed 30-fold dilution of serum in the blank reagent. The blank reagent consisted of high purity water (>18 MΩ), TMAH (AlfaAesar, Ward Hill, MA, USA), Triton X-100 (PerkinElemer, Waltham, MA, USA), n-butanol (Merck, Munich, Germany), and EDTA (Sigma Aldrich, Burlington, MA, USA).

### 2.3. Quality Control

The accuracy and precision of measurements were tested using certified reference material (CRM), Clincheck Plasmonorm Serum Trace Elements Level 1 (Recipe, Munich, Germany) Each patient in the study completed an EC and lifestyle risk factors form.

### 2.4. Statistics

All of the patients included in the study were assigned to one of three groups (TI-TIII tertiles), determined on the basis of the distribution of Cu and Zn levels in the blood serum. The division into tertiles was connected with the analysis of the Cu/Zn ratio, and aimed at assessing whether a protective effect could be observed for balanced values of this parameter. The patients were analyzed in terms of levels of Zn (TI-TIII), Cu (TI-TIII), and Cu/Zn ratio (TI-TIII) and factors such as BMI, age of first menstruation, number of deliveries, breastfeeding (yes/no), menopause (yes/no), smoking (yes/no), contraception (yes/no), menopausal hormone therapy (yes/no), diabetes (yes/no), hypertension (yes/no), endometriosis (yes/no), and hypothyroidism (yes/no). The Cu, Zn, and Cu/Zn ratio tertiles with the lowest number of EC patients were considered as the reference subgroups in further analyses (TIII, TIII, and TII, respectively).

To estimate the association of the aforementioned factors with EC occurrence, odds ratios (OR) and 95% confidence intervals (CIs) were calculated using univariable and multivariable conditional logistic regression. Significant differences between the analyzed groups were identified at *p* < 0.05 All of the statistical calculations were performed using R: A language and environment for statistical computing. R Foundation for Statistical Computing, Vienna, Austria; https://www.R-project.org/, accessed on 31 October 2022 (R version 4.2.2).

The characteristics of the study population are presented in Table 1.

## 3. Results

The mean level of Zn for all patients was 804.36 μg/L, while in the study group, the average level was 707.05 μg/L (144.94–1436.25) The mean level of Zn for the control group was 901.67 μg/L (393.71–2154.51). The mean serum Cu level was 1067.91 μg/L (136.21–2373.72) among all women included in the study, and 959.39 μg/L and 1176.42 μg/L in the diseased and control groups, respectively. The detailed percentage distribution of diseased women and the control group in particular tertiles, as well as the corresponding ranges of Zn and Cu concentrations, are presented in Table 1. For both Cu and Zn, in the univariable and multivariable analysis, an increased incidence of EC was observed in the groups of women with lower serum levels of these elements. In the analysis for Zn, the first and the second tertiles had a higher incidence of EC, and the OR was 15.0 (95% CI 6.54–34.5; *p* < 0.001) for the first tertile and 3.67 (95% CI 1.79–7.54; *p* < 0.001) for the second tertile in the univariable analysis and 23.2 (95% CI 6.92–78.0; *p* < 0.001) and 4.81 (95% CI 1.75–13.3; *p* < 0.001) in the multivariable analysis, respectively. The results are presented in Table 2.

When analyzing the Cu level in the serum, a lower concentration of this trace element was associated with a higher incidence of EC. For tertile I (with the lowest Cu level), the obtained OR was 8.54 (95% CI 4.14–17.6; *p* < 0.001) in the univariable analysis and 13.9 (95% CI 4.96–38.8; *p* < 0.001) in the multivariable analysis. For tertile II, the odds ratio was 3.33 (95% CI 1.69–6.57; *p* < 0.001) in the univariable analysis and 7.49 (95% CI 2.61–21.5; *p* < 0.001) in the multivariable analysis. The results of the analysis for the entire population are presented in Table 3.

In order to analyze the relationship between the Cu/Zn ratio and the occurrence of EC in the study population, three groups were distinguished on the basis of the Cu/Zn ratio value. The ranges for the tertiles were: 0.46–1.20 (mean 1.01) for tertile I, 1.20–1.46 (mean 1.34) for tertile II, and 1.47–3.18 (mean 1.77) for tertile III. Tertile II, presenting the middle values of the Cu/Zn ratio in the study population and containing 39% of patients from the control group and 27% of patients from the study group, was taken as the reference. The results indicate the lowest occurrence of EC in the reference tertile, with statistical significance achieved for tertile III with the highest Cu/Zn ratio. The resulting OR was 2.21 (95% CI 1.25–3.89; *p* = 0.004). The results of the analysis are presented in Table 4.

For the remaining analyzed factors, the results for BMI, breastfeeding, number of deliveries, diabetes, and menopausal hormone therapy were statistically significant in the univariable analysis.

## 4. Discussion

In our study, the results obtained for both Cu and Zn indicated an increase in the occurrence of EC with a decrease in the tested microelements levels. The patients with EC were characterized by lower serum levels of Cu and Zn compared with the age-matched control group. Despite the fact that studies on the impact of micronutrient levels on the risk of malignant tumors are gaining interest from many researchers, a review of the available literature shows few items regarding the relationship between Cu and Zn levels and the occurrence of EC.

In their study, Atakul et al. reported a similar correlation between Cu and Zn levels and the Cu/Zn ratio and EC. The study included 47 patients with EC and 45 patients from the control group. The study group had a lower mean level of Cu and Zn compared with the control group (*p* < 0.001) [83]. Michalczyk et al. analyzed the serum levels of Cu, Zn, Fe, and Mn among 110 women with different endometrial pathologies. The study group included 21 patients with endometrial cancer. They were characterized by the lowest serum Zn level and a higher median level of Cu. The median serum Cu/Zn ratio was also higher in the endometrial cancer patients when compared with the patients with endometrial polyps or normal endometrial tissue. However, the differences were not big enough to reach statistical significance [84]. In the study by Margalioth et al., the patients diagnosed with EC were characterized by a higher level of Cu than the patients from the control group (152 ± 6 μg/dL vs. 126 ± 4 μg/dL) [85]. In the mendelian randomization study, Wang et al. observed that the genetically predicted Cu level is a factor associated with EC risk, especially for risk of endometrioid endometrial cancer (OR 1.17; *p* = 0.04) [86]. It has to be pointed, as the authors noted, that sensitivity analyses could not be performed because only two variants were included in the genetic instrument. Thus, a larger GWAS for Cu levels is required. There was no association between Zn level and EC in this study. In a recent study, Zhu et al. found, after adjustment for potential confounders, a positive correlation between Cu intake and EC (*p* = 0.009). There was no correlation between Zn intake and EC; however, there was a negative correlation between Zn intake and ovarian cancer [87]. Yaman et al. examined the tissue samples of patients with EC and ovarian cancer (OC) and healthy ones in order to compare the levels of trace elements in them [88]. As a result of the study, there were no significant differences in Cu content in the EC group and in the group of benign lesions, while for Zn, the levels in EC tissues were lower (*p* = 0.005). Different results were presented in the study by Nasiadek et al., where no differences in tissue levels were noted [89]. This difference may have resulted from the design of the study, where the tissues came from the same patient from undisturbed sites.

For other malignancies of female genital organs, the available research results are often contradictory. Cuzhi et al. examined the levels of ions (Cu, Zn, Fe, Mn, Ca, and Se) in the tissues and sera of patients with cervical cancer and fibroids compared with the control group, and found a lower concentration of Zn in the study group, while for Cu and Cu/Zn ratio, these results were opposite [90]. Zhang et al., in their meta-analysis, reported higher Cu concentrations in cervical cancer patients compared with the control group (*p* < 0.001) [91].

A similar behavior of the Cu and Cu/Zn ratio was noted by Marinov et al. The study included patients with benign and malignant ovarian tumors compared with the group without ovarian diseases. In both groups with ovarian lesions, both the Cu level and the Cu/Zn ratio were elevated. The Cu levels for ovarian cancer patients were 22.98 ± 3.90 µmol/L), and for the benign lesions they were 18.40 ± 4.61 µmol/L (*p* < 0.05) compared with the Cu levels in the control group. The Cu/Zn ratio followed the same trend, 1.68 and 1.42, respectively [92]. The same author, examining the concentration of Zn in an identically constructed study, did not find any differences in the content of this element in the sera of all of the studied groups [93].

There are papers whose authors have studied the relationship between Cu and Zn and cancers of other locations. The most frequently studied cancers are prostate cancer, colorectal cancer, bladder cancer, thyroid cancer, and breast cancer. Mao and Huang conducted a systematic review of six studies on Cu and Zn content in the sera and urine of bladder cancer patients compared with the control group [94]. The patients with bladder cancer showed lower serum Zn levels (*p* < 0.001) and higher Cu levels (*p* = 0.006). The same conclusions were obtained in a clinical study of patients with prostate cancer by Golabek et al. [95]. A meta-analysis by Feng et al. of 36 studies showed identical trends for breast cancer (*p* < 0.001) [96]. The patients with breast cancer had low Zn levels and higher Cu and Cu/Zn ratio compared with the control group. In a case-control study related to The European Prospective Investigation into Cancer and Nutrition (EPIC), Stępień et al. showed a similar behavior of the described trace elements in patients with colorectal cancer (CRC) [97]. Higher Cu levels increased the risk of CRC (OR = 1.50; 95% CI: 1.06–2.13; *p* = 0.02), while high Zn levels were associated with a lower risk (OR = 0.65; 95% CI: 0.43, 0.97; *p* = 0.07). Consequently, the ratio of Cu/Zn was positively associated with CRC (OR = 1.70; 95% CI: 1.20–2.40; *p* = 0.0005). These results were not confirmed by Zhang et al.’s analysis based on the results of the National Health and Nutrition Examination 2011–2016 [74]. The authors did not find a significant effect of Cu and Zn on the risk of developing CRC. The only observation consistent with the previously mentioned study was an increased risk of CRC with a higher Cu/Zn ratio.

The limitations of our study include the lack of information on the dietary factors among the surveyed women. Therefore, it is not possible to assess the impact of diet on the obtained results, taking into account both the intake of the studied microelements and general dietary habits related to macroelements. The results from the available studies suggest a relationship between diet and Cu and Zn serum levels [98,99,100,101]. It should also be noted that the cancer process may be a factor that influences the levels of the tested microelements. There is some evidence of increased Cu concentrations in cancer cells and changes in serum Cu concentrations associated with response to cancer treatment, which may support the theory of the influence of the cancer itself on the metabolism of this microelement [102,103]. Prospective studies are required to distinguish the effects of cancer on Cu and Zn serum levels from pre-morbid changes.

It should be noted here that observed behavior of the serum Cu level in EC patients differs from changes occurring in other cancers. Both our study and that of Atakul et al. suggest a relationship between the occurrence of EC and reduced Cu levels. The discussed studies on malignant neoplasms of other locations show the opposite relationship: the higher the serum Cu level, the more frequent the occurrence of malignant neoplasms. It can be assumed that the observations of the relationship between the level of Cu and EC were in agreement with the results of studies suggesting a protective effect of Cu-releasing intrauterine devices on the risk of EC [104,105,106].

The differences in the results of the presented studies may also result from the fact that the levels of Cu and Zn are influenced by various environmental factors. Johnson et al., in a study of 127 women and men, found significantly higher levels of Cu and ceruloplasmin in the serum of women (*p* < 0.005) [107]. A correlation in the levels of these micronutrients with age was also found: in older people, the level of Cu increased and the level of Zn decreased [108]. Moreover, low levels of Zn have been observed in people with age-dependent diseases (Parkinson’s and Alzheimer’s) [109]. In addition, the levels of these micronutrients may be influenced by hormonal factors. Hormone therapy, without affecting the level of Zn, contributes to the increase in the concentration of Cu in the body [110]. Michos et al. studied changes in the level of the described ions in the serum of patients during the menstrual cycle. The results obtained by the researchers showed a positive relationship between estrogen and Zn levels, and a negative relationship with Cu (*p* < 0.05) [111]. It should be noted that the micronutrients themselves can affect each other’s levels in the body. Excessive levels of Zn block the intestinal absorption of Cu, leading to hypocupremia [112].

## 5. Conclusions

The obtained results indicate the existence of a correlation between the occurrence of EC and low levels of Cu and Zn in the serum. The patients from the study group had lower levels of both Cu and Zn compared with patients from the control group. Women with lower Cu and Zn serum levels may contribute to the group with higher risk of EC occurrence. Particularly interesting were the observed differences in Cu levels between diseased women and the control group, which differed from the results of most available studies for other cancers. The obtained results indicated a lower occurrence of endometrial cancer in patients with a specific balance between serum Cu and Zn level (Cu/Zn ratio in the range of 1.20–1.46). Because of the described participation of these ions in carcinogenesis, this seems to be an interesting direction for further research. Prospective population studies are needed to determine whether the levels of the micronutrients tested and their ratio may be helpful for identifying a group at increased risk of developing EC.

## Figures and Tables

**Table 1:** Characteristics of the population included in the study.

Variables	Overall, n = 306	Control, n = 153	Diseased, n = 153	*p*-Value
Micronutrients in serum				
Zn [μg/L]	144.94–2154.51 (804.36)	393.71–2154.51 (901.67)	144.94–1436.25 (707.05)	
I tertile 144.94–688.20 (580.27 ± 97.26)	101 (33%)	19 (12%)	82 (54%)	<0.001
II tertile 688.76–892.91 (786.85 ± 59.72)	101 (33%)	52 (34%)	49 (32%)	<0.001
III tertile (reference) 894.33–2154.51 (1039.00 ± 163.06)	104 (34%)	82 (54%)	22 (14%)	
Cu [μg/L]	136.21–2373.72 (1067.91)	499.30–2373.72 (1176.42)	136.21–1504.92 (959.39)	
I tertile 136.21–963.33 (785.41 ± 154.65)	101 (33%)	25 (16%)	76 (50%)	<0.001
II tertile 966.10–1176.60 (1068.81 ± 61.07)	101 (33%)	50 (33%)	51 (33%)	<0.001
III tertile (reference) 1178.85–2373.72 (1341.38 ± 159.97)	104 (34%)	78 (51%)	26 (17%)	
Cu/Zn ratio				
I tertile 0.46–1.20 (1.01 ± 0.14)	101 (33%)	52 (34%)	49 (32%)	0.3
II tertile (reference) 1.20–1.46 (1.34 ± 0.08)	101 (33%)	60 (39%)	41 (27%)	
III tertile 1.47–3.18 (1.77 ± 0.30)	104 (34%)	41 (27%)	63 (41%)	0.006
Health history				
BMI	18.03–56.50 (29.38)	18.03–43.51 (27.50)	19.63–56.50 (31.27)	<0.001
Smoking				
No	272 (89%)	140 (92%)	132 (86%)	
Yes	34 (11%)	13 (8.5%)	21 (14%)	0.2
Diabetes				
No	249 (81%)	133 (87%)	116 (76%)	
Yes	57 (19%)	20 (13%)	37 (24%)	0.014
Hypertension				
No	132 (43%)	73 (48%)	59 (39%)	
Yes	174 (57%)	80 (52%)	94 (61%)	0.073
Hypothyroidism				
No	261 (85%)	130 (85%)	131 (86%)	
Yes	45 (15%)	23 (15%)	22 (14%)	0.9
Gynecology history				
Age of first menstruation	10.00–20.00 (14.16)	11.00–20.00 (14.16)	10.00–18.00 (14.46)	>0.9
Number of deliveries	0.00–8.00 (2.61)	0.00–8.00 (2.77)	0.00–7.00 (2.44)	0.033
Breastfeeding				
No	78 (25%)	26 (17%)	52 (34%)	
Yes	228 (75%)	127 (83%)	101 (66%)	0.002
Menopause				
No	24 (7.8%)	13 (8.5%)	11 (7.2%)	
Yes	282 (92%)	140 (92%)	142 (93%)	0.5
Contraception				
No	286 (93%)	140 (92%)	146 (95%)	
Yes	20 (6.5%)	13 (8.5%)	7 (4.6%)	0.12
Menopausal hormone therapy				
No	283 (92%)	148 (97%)	135 (88%)	
Yes	23 (7.5%)	5 (3.3%)	18 (12%)	0.011
Endometriosis				
No	272 (89%)	141 (92%)	131 (86%)	
Yes	34 (11%)	12 (7.8%)	22 (14%)	0.074

**Table 2:** Results of Zn serum level analysis for the whole population.

	Univariable Conditional Logistic Regression			Multivariable Conditional Logistic Regression		
Variables	OR	95% CI	*p*-Value	OR	95% CI	*p*-Value
Zn						
I tertile 144.94–688.20 (580.27 ± 97.26)	15.0	6.54–34.5	<0.001	23.2	6.92–78.0	<0.001
II tertile 688.76–892.91 (786.85 ± 59.72)	3.67	1.79–7.54	<0.001	4.81	1.75–13.3	0.002
III (reference) 894.33–2154.51 (1039.00 ± 163.06)	—	—		—	—	
BMI	1.15	1.08–1.21	<0.001	1.16	1.06–1.26	<0.001
Smoking						
No	—	—		—	—	
Yes	1.67	0.81–3.41	0.2	0.67	0.18–2.53	0.6
Diabetes						
No	—	—		—	—	
Yes	2.21	1.18–4.16	0.014	2.05	0.69–6.07	0.2
Hypertension						
No	—	—		—	—	
Yes	1.61	0.96–2.71	0.073	1.36	0.59–3.12	0.5
Hypothyroidism						
No	—	—		—	—	
Yes	0.94	0.49–1.83	0.9	1.70	0.54–5.29	0.4
Age of first menstruation	1.00	0.87–1.15	>0.9	1.12	0.88–1.41	0.4
Number of deliveries	0.82	0.69–0.99	0.033	0.89	0.66–1.20	0.4
Breastfeeding						
No	—	—		—	—	
Yes	0.42	0.25–0.72	0.002	0.31	0.12–0.76	0.011
Menopause						
No	—	—		—	—	
Yes	1.67	0.40–6.97	0.5	0.26	0.04–1.85	0.2
Contraception						
No	—	—		—	—	
Yes	0.40	0.13–1.28	0.12	1.07	0.19–6.15	>0.9
Menopausal hormone therapy						
No	—	—		—	—	
Yes	3.60	1.34–9.70	0.011	1.84	0.41–8.21	0.4
Endometriosis						
No	—	—		—	—	
Yes	2.00	0.94–4.27	0.074	4.12	1.03–16.4	0.045

**Table 3:** Results of the Cu serum level analysis for the whole population.

	Univariable Conditional Logistic Regression			Multivariable Conditional Logistic Regression		
Variables	OR	95% CI	*p*-Value	OR	95% CI	*p*-Value
Cu						
I tertile 136.21–963.33 (785.41 ± 154.65)	8.54	4.14–17.6	<0.001	13.9	4.96–38.8	<0.001
II tertile 966.10–1176.60 (1068.81 ± 61.07)	3.33	1.69–6.57	<0.001	7.49	2.61–21.5	<0.001
III tertile (reference) 1178.85–2373.72 (1341.38 ± 159.97)	—	—		—	—	
BMI	1.15	1.08–1.21	<0.001	1.23	1.12–1.35	<0.001
Smoking						
No	—	—		—	—	
Yes	1.67	0.81–3.41	0.2	1.38	0.45–4.29	0.6
Diabetes						
No	—	—		—	—	
Yes	2.21	1.18–4.16	0.014	1.93	0.73–5.09	0.2
Hypertension						
No	—	—		—	—	
Yes	1.61	0.96–2.71	0.073	0.90	0.39–2.07	0.8
Hypothyroidism						
No	—	—		—	—	
Yes	0.94	0.49–1.83	0.9	0.96	0.29–3.24	>0.9
Age of first menstruation	1.00	0.87–1.15	>0.9	1.11	0.89–1.39	0.4
Number of deliveries	0.82	0.69–0.99	0.033	0.91	0.68–1.22	0.5
Breastfeeding						
No	—	—		—	—	
Yes	0.42	0.25–0.72	0.002	0.30	0.12–0.75	0.010
Menopause						
No	—	—		—	—	
Yes	1.67	0.40–6.97	0.5	1.53	0.19–12.7	0.7
Contraception						
No	—	—		—	—	
Yes	0.40	0.13–1.28	0.12	0.51	0.08–3.24	0.5
Menopause hormonal therapy						
No	—	—		—	—	
Yes	3.60	1.34–9.70	0.011	2.07	0.42–10.2	0.4
Endometriosis						
No	—	—		—	—	
Yes	2.00	0.94–4.27	0.074	4.12	1.15–14.7	0.029

**Table 4:** Results of the Cu/Zn ratio analysis in the study population.

	Univariable Conditional Logistic Regression			Multivariable Conditional Logistic Regression		
Variables	OR	95% CI	*p*-Value	OR	95% CI	*p*-Value
Cu/Zn						
I tertile 0.46–1.20 (1.01 ± 0.14)	1.38	0.78–2.43	0.3	1.69	0.79–3.62	0.2
II tertile (reference) 1.20–1.46 (1.34 ± 0.08)	—	—		—	—	
III tertile 1.47–3.18 (1.77 ± 0.30)	2.21	1.25–3.89	0.006	1.86	0.88–3.91	0.10
BMI	1.15	1.08–1.21	<0.001	1.18	1.10–1.27	<0.001
Smoking						
No	—	—		—	—	
Yes	1.67	0.81–3.41	0.2	1.75	0.68–4.51	0.2
Diabetes						
No	—	—		—	—	
Yes	2.21	1.18–4.16	0.014	1.27	0.56–2.87	0.6
Hypertension						
No	—	—		—	—	
Yes	1.61	0.96–2.71	0.073	1.33	0.68–2.61	0.4
Hypothyroidism						
No	—	—		—	—	
Yes	0.94	0.49–1.83	0.9	1.28	0.49–3.37	0.6
Age of first menstruation	1.00	0.87–1.15	>0.9	1.16	0.95–1.41	0.14
Number of deliveries	0.82	0.69–0.99	0.033	0.95	0.74–1.22	0.7
Breastfeeding						
No	—	—		—	—	
Yes	0.42	0.25–0.72	0.002	0.40	0.19–0.84	0.016
Menopause						
No	—	—		—	—	
Yes	1.67	0.40–6.97	0.5	0.41	0.07–2.41	0.3
Contraception						
No	—	—		—	—	
Yes	0.40	0.13–1.28	0.12	0.39	0.09–1.76	0.2
Menopause hormonal therapy						
No	—	—		—	—	
Yes	3.60	1.34–9.70	0.011	4.94	1.31–18.6	0.018
Endometriosis						
No	—	—		—	—	
Yes	2.00	0.94–4.27	0.074	2.62	0.89–7.66	0.080

## Data Availability

The data presented in this study are available upon request from the corresponding authors.
